# Genome-wide interaction analysis of folate for colorectal cancer risk

**DOI:** 10.1016/j.ajcnut.2023.08.010

**Published:** 2023-08-26

**Authors:** Emmanouil Bouras, Andre E. Kim, Yi Lin, John Morrison, Mengmeng Du, Demetrius Albanes, Elizabeth L. Barry, James W. Baurley, Sonja I. Berndt, Stephanie A. Bien, Timothy D. Bishop, Hermann Brenner, Arif Budiarto, Andrea Burnett-Hartman, Peter T. Campbell, Robert Carreras-Torres, Graham Casey, Tjeng Wawan Cenggoro, Andrew T. Chan, Jenny Chang-Claude, David V. Conti, Michelle Cotterchio, Matthew Devall, Virginia Diez-Obrero, Niki Dimou, David A. Drew, Jane C. Figueiredo, Graham G. Giles, Stephen B. Gruber, Marc J. Gunter, Tabitha A. Harrison, Akihisa Hidaka, Michael Hoffmeister, Jeroen R. Huyghe, Amit D. Joshi, Eric S. Kawaguchi, Temitope O. Keku, Anshul Kundaje, Loic Le Marchand, Juan Pablo Lewinger, Li Li, Brigid M. Lynch, Bharuno Mahesworo, Satu Männistö, Victor Moreno, Neil Murphy, Polly A. Newcomb, Mireia Obón-Santacana, Jennifer Ose, Julie R. Palmer, Nikos Papadimitriou, Bens Pardamean, Andrew J. Pellatt, Anita R. Peoples, Elizabeth A. Platz, John D. Potter, Lihong Qi, Conghui Qu, Gad Rennert, Edward Ruiz-Narvaez, Lori C. Sakoda, Stephanie L. Schmit, Anna Shcherbina, Mariana C. Stern, Yu-Ru Su, Catherine M. Tangen, Duncan C. Thomas, Yu Tian, Caroline Y. Um, Franzel JB. van Duijnhoven, Bethany Van Guelpen, Kala Visvanathan, Jun Wang, Emily White, Alicja Wolk, Michael O. Woods, Cornelia M. Ulrich, Li Hsu, W James Gauderman, Ulrike Peters, Konstantinos K. Tsilidis

**Affiliations:** 1 Department of Hygiene and Epidemiology, University of Ioannina School of Medicine, Ioannina, Greece; 2 Division of Biostatistics, Department of Population and Public Health Sciences, Keck School of Medicine, University of Southern California, Los Angeles, CA, United States; 3 Public Health Sciences Division, Fred Hutchinson Cancer Center, Seattle, WA, United States; 4 Department of Epidemiology and Biostatistics, Memorial Sloan Kettering Cancer Center, New York, NY, United States; 5 Division of Cancer Epidemiology and Genetics, National Cancer Institute, National Institutes of Health, Bethesda, MD, United States; 6 Department of Epidemiology, Geisel School of Medicine at Dartmouth, Hanover, NH, United States; 7 Bioinformatics and Data Science Research Center, Bina Nusantara University, Jakarta, Indonesia; 8 BioRealm LLC, Walnut, CA, United States; 9 Leeds Institute of Cancer and Pathology, University of Leeds, Leeds, United Kingdom; 10 Division of Clinical Epidemiology and Aging Research, German Cancer Research Center (DKFZ), Heidelberg, Germany; 11 Division of Preventive Oncology, German Cancer Research Center (DKFZ) and National Center for Tumor Diseases (NCT), Heidelberg, Germany; 12 German Cancer Consortium (DKTK), German Cancer Research Center (DKFZ), Heidelberg, Germany; 13 Computer Science Department, School of Computer Science, Bina Nusantara University, Jakarta, Indonesia; 14 Institute for Health Research, Kaiser Permanente Colorado, Denver, CO, United States; 15 Department of Epidemiology and Population Health, Albert Einstein College of Medicine, Bronx, NY, United States; 16 Unit of Biomarkers and Suceptibility (UBS), Oncology Data Analytics Program (ODAP), Catalan Institute of Oncology (ICO), L’Hospitalet del Llobregat, Barcelona, Spain; 17 ONCOBELL Program, Bellvitge Biomedical Research Institute (IDIBELL), L’Hospitalet de Llobregat, Barcelona, Spain; 18 Digestive Diseases and Microbiota Group, Girona Biomedical Research Institute (IDIBGI), Salt, Girona, Spain; 19 Center for Public Health Genomics, University of Virginia, Charlottesville, VA, United States; 20 Division of Gastroenterology, Massachusetts General Hospital and Harvard Medical School, Boston, MA, United States; 21 Channing Division of Network Medicine, Brigham and Women’s Hospital and Harvard Medical School, Boston, MA, United States; 22 Clinical and Translational Epidemiology Unit, Massachusetts General Hospital and Harvard Medical School, Boston, MA, United States; 23 Broad Institute of Harvard and MIT, Cambridge, MA, United States; 24 Department of Epidemiology, Harvard TH Chan School of Public Health, Harvard University, Boston, MA, United States; 25 Department of Immunology and Infectious Diseases, Harvard TH Chan School of Public Health, Harvard University, Boston, MA, United States; 26 Division of Cancer Epidemiology, German Cancer Research Center (DKFZ), Heidelberg, Germany; 27 University Medical Centre Hamburg-Eppendorf, University Cancer Centre Hamburg (UCCH), Hamburg, Germany; 28 Ontario Health (Cancer Care Ontario), Toronto, Ontario, Canada; 29 Center for Public Health Genomics, Department of Public Health Sciences, University of Virginia, Charlottesville, VA, United States; 30 Department of Public Health Sciences, Center for Public Health Genomics, Charlottesville, VA, United States; 31 Nutrition and Metabolism Branch, International Agency for Research on Cancer, Lyon, France; 32 Department of Medicine, Samuel Oschin Comprehensive Cancer Institute, Cedars-Sinai Medical Center, Los Angeles, CA, United States; 33 Cancer Epidemiology Division, Cancer Council Victoria, Melbourne, Victoria, Australia; 34 Centre for Epidemiology and Biostatistics, Melbourne School of Population and Global Health, The University of Melbourne, Melbourne, Victoria, Australia; 35 Precision Medicine, School of Clinical Sciences at Monash Health, Monash University, Clayton, VIC, Australia; 36 Department of Medical Oncology & Therapeutics Research, City of Hope National Medical Center, Duarte, CA, United States; 37 Department of Biostatistics, Fielding School of Public Health, University of California, Los Angeles, Los Angeles, CA, United States; 38 Center for Gastrointestinal Biology and Disease, University of North Carolina, Chapel Hill, NC, United States; 39 Department of Genetics, Stanford University, Stanford, CA, United States; 40 Department of Computer Science, Stanford University, Stanford, CA, United States; 41 University of Hawaii Cancer Center, Honolulu, HI, United States; 42 Department of Family Medicine, University of Virginia, Charlottesville, VA, United States; 43 Cancer Epidemiology Division, Cancer Council Victoria, Melbourne, VIC, Australia; 44 Department of Public Health Solutions, National Institute for Health and Welfare, Helsinki, Finland; 45 Consortium for Biomedical Research in Epidemiology and Public Health (CIBERESP), Madrid, Spain; 46 Department of Clinical Sciences, Faculty of Medicine and health Sciences and Universitat de Barcelona Institute of Complex Systems (UBICS), University of Barcelona (UB), L’Hospitalet de Llobregat, Barcelona, Spain; 47 School of Public Health, University of Washington, Seattle, WA, United States; 48 Huntsman Cancer Institute, University of Utah, Salt Lake City, Utah; 49 Slone Epidemiology Center at Boston University, Boston, MA, United States; 50 Department of Cancer Medicine, MD Anderson Cancer Center, Houston, TX, United States; 51 Department of Epidemiology, Johns Hopkins Bloomberg School of Public Health, Baltimore, MD, United States; 52 Research Centre for Hauora and Health, Massey University, Wellington, New Zealand; 53 Department of Public Health Sciences, University of California Davis, Davis, CA, United States; 54 Department of Community Medicine and Epidemiology, Lady Davis Carmel Medical Center, Haifa, Israel; 55 Ruth and Bruce Rappaport Faculty of Medicine, Technion-Israel Institute of Technology, Haifa, Israel; 56 Clalit National Cancer Control Center, Haifa, Israel; 57 Department of Nutritional Sciences, University of Michigan School of Public Health, Ann Arbor, MI, United States; 58 Division of Research, Kaiser Permanente Northern California, Oakland, CA, United States; 59 Genomic Medicine Institute, Cleveland Clinic, Cleveland, OH, United States; 60 Population and Cancer Prevention Program, Case Comprehensive Cancer Center, Cleveland, OH, United States; 61 Department of Population and Public Health Sciences and Norris Comprehensive Cancer Center, Preventive Medicine, Keck School of Medicine, University of Southern California, Los Angeles, CA, United States; 62 SWOG Statistical Center, Fred Hutchinson Cancer Research Center, Seattle, WA, United States; 63 School of Public Health, Capital Medical University, Beijing, China; 64 Department of Population Science, American Cancer Society, Atlanta, GA, United States; 65 Division of Human Nutrition and Health, Wageningen University & Research, Wageningen, The Netherlands; 66 Department of Radiation Sciences, Oncology Unit, Umeå University, Umeå, Sweden; 67 Wallenberg Centre for Molecular Medicine, Umeå University, Umeå, Sweden; 68 Department of Epidemiology, University of Washington School of Public Health, Seattle, WA, United States; 69 Institute of Environmental Medicine, Karolinska Institutet, Stockholm, Sweden; 70 Memorial University of Newfoundland, Discipline of Genetics, St John’s, Canada; 71 Department of Biostatistics, University of Washington, Seattle, WA, United States; 72 Department of Epidemiology, University of Washington, Seattle, WA, United States; 73 Department of Epidemiology and Biostatistics, Imperial College London, School of Public Health, London, United Kingdom; 74 Department of Population Health Sciences, University of Utah, Salt Lake City, UT, United States

**Keywords:** folate, folic acid, colorectal cancer, CRC, genome-wide, interaction, GWIS, European, SYN2, synapsin, TIMP4, tissue inhibitor of metalloproteinase 4

## Abstract

**Background:**

Epidemiological and experimental evidence suggests that higher folate intake is associated with decreased colorectal cancer (CRC) risk; however, the mechanisms underlying this relationship are not fully understood. Genetic variation that may have a direct or indirect impact on folate metabolism can provide insights into folate’s role in CRC.

**Objectives:**

Our aim was to perform a genome-wide interaction analysis to identify genetic variants that may modify the association of folate on CRC risk.

**Methods:**

We applied traditional case-control logistic regression, joint 3-degree of freedom, and a 2-step weighted hypothesis approach to test the interactions of common variants (allele frequency >1%) across the genome and dietary folate, folic acid supplement use, and total folate in relation to risk of CRC in 30,550 cases and 42,336 controls from 51 studies from 3 genetic consortia (CCFR, CORECT, GECCO).

**Results:**

Inverse associations of dietary, total folate, and folic acid supplement with CRC were found (odds ratio [OR]: 0.93; 95% confidence interval [CI]: 0.90, 0.96; and 0.91; 95% CI: 0.89, 0.94 per quartile higher intake, and 0.82 (95% CI: 0.78, 0.88) for users compared with nonusers, respectively). Interactions (*P-*interaction < 5×10^-8^) of folic acid supplement and variants in the 3p25.2 locus (in the region of Synapsin II [*SYN2*]/tissue inhibitor of metalloproteinase 4 [*TIMP4*]) were found using traditional interaction analysis, with variant rs150924902 (located upstream to *SYN2*) showing the strongest interaction. In stratified analyses by rs150924902 genotypes, folate supplementation was associated with decreased CRC risk among those carrying the TT genotype (OR: 0.82; 95% CI: 0.79, 0.86) but increased CRC risk among those carrying the TA genotype (OR: 1.63; 95% CI: 1.29, 2.05), suggesting a qualitative interaction (*P*-interaction = 1.4×10^-8^). No interactions were observed for dietary and total folate.

**Conclusions:**

Variation in 3p25.2 locus may modify the association of folate supplement with CRC risk. Experimental studies and studies incorporating other relevant omics data are warranted to validate this finding.

## Introduction

Colorectal cancer (CRC) represents a major public health concern, being the third most common cancer worldwide with nearly 2 million incident cases and the second cause of cancer death in 2020 [1]. Human diet, being a source of substances with heterogeneous effects that have the potential to alter colonocyte metabolism and affect tumorigenesis, is particularly important for CRC risk [2]. Considering its functional roles, folate has gained considerable attention over the years in the field of CRC research [3]. Folate contributes to DNA biosynthesis, repair and methylation, and key processes in cellular homeostasis, with direct implications in terms of carcinogenesis. Experimental evidence supports the preventive effect of folic acid in carcinogenesis; nevertheless, it has been postulated that folic acid may have a dual role in normal and neoplastic colorectal tissues and that excess folate might enhance the progression of already existing premalignant and malignant lesions [[4], [5], [6], [7]]. Epidemiological studies generally report inverse associations of folate intake with CRC, whereas studies of circulating folate concentrations have found mixed associations [[8], [9], [10], [11], [12], [13], [14]]. Furthermore, it has been suggested that a latency period exists for folate intake, beyond which no benefit is observed [15].

Until recently, more than 200 common genetic variants have been identified in genome-wide association studies (GWASs) of CRC risk, with a total contribution of common variants to the familial risk estimated to be nearly 20% [[16], [17], [18], [19], [20]]. Investigating the interaction between genetic variants and environmental factors may lead to the unveiling of novel genetic loci and capture part of the missing heritability [21]. Previous studies were limited to a few single nucleotide polymorphisms (SNPs) in candidate genes in the folate-mediated one-carbon metabolism (FOCM) pathway and have largely shown inconsistent interactions [[22], [23], [24], [25], [26], [27], [28]].

Gene-by-folate interaction may explain some of the observed inconsistencies and can add biological insights on the role of folate in colorectal carcinogenesis. We therefore performed a genome-wide interaction analysis to identify SNPs that may modify the effects of dietary and total folate and folic acid supplementation on CRC risk. Moreover, by pooling data from 51 studies in the largest sample available to date, we provide the most robust estimate on the marginal associations of folate with CRC risk.

## Methods

### Study participants

We included data from a total of 30,550 cases and 42,336 controls from 51 studies contributing to 3 consortia—the multicentered Colon Cancer Family Registry (CCFR), the Genetics and Epidemiology of Colorectal Cancer Consortium (GECCO), and the Colorectal Transdisciplinary Study (CORECT) (Supplemental Table 1 and Supplemental Figure 1). Controls were matched on age, sex, race/ethnicity, and enrollment date/trial group, when applicable. Cases were defined as colorectal adenocarcinoma or advanced adenomas (defined as an adenoma 1 cm or larger in diameter and/or with tubulovillous, villous, or high-grade dysplasia/carcinoma-in-situ histology—including 2474 cases with advanced adenomas) and were confirmed by medical records, pathological reports, or death certificate information. For the small subset of advanced adenoma cases, matched controls were polyp-free as displayed via sigmoidoscopy or colonoscopy at the time of adenoma selection. All participants gave written informed consent, and the studies were approved by their respective Institutional Review Boards. Description of the study design and details on the GWAS has been previously described [27,29].

### Exposure assessment

Interviews and/or structured questionnaires were used to obtain information on demographics and environmental risk factors. A multistep data harmonization procedure was carried out at the GECCO coordinating center (Fred Hutchinson Cancer Research Center), reconciling each study’s unique protocols and data collection instruments as described previously [[30], [31], [32]]. Dietary folate was estimated within each study, at the reference time (usually the time period 1 to 2 y prior to diagnosis or selection for case-control studies and at time of enrollment or blood collection for cohort studies), by linking items from food frequency questionnaires or diet history, with nutrient databases, accounting for folate fortification when applicable (e.g., information collected in US studies after the year 1998). Folate and folic acid intake in each study was determined based on micrograms per day (μg/d) of folate from foods (i.e., dietary folate) or supplements (single or multivitamins). Supplemental folate intake was estimated using actual quantities when available, otherwise assumed to be doses of 400 μg/d. To account for the higher bioavailability of synthetic folic acid compared with natural food folate, we calculated total folate intake as dietary folate equivalents (total μg dietary folate equivalents [DFE] = μg of dietary folate + 1.7 × μg folic acid from supplements) [33]. Because the times of enrollment for Prostate, Lung, Colorectal, & Ovarian Cancer Screening Trial (PLCO), VITamin And Lifestyle Study, and Women’s Health Initiative (WHI) overlapped or followed the period of folic acid fortification (1996–1998), these studies accounted for folic acid fortification when calculating dietary folate intake and entered dietary folate intake as μg of natural food folate + 1.7 × μg folic acid from fortified food. We performed separate analyses considering folic acid supplement intake as a binary variable (yes/no). Similarly, food frequency questionnaires and diet histories were used to ascertain diet-related exposures and total energy consumption (kcal/d) at the reference time. The harmonized alcohol intake variable was expressed as grams per day and categorized into 2 groups: nondrinkers (≤1 g/d) and drinkers (>1 g/d). Standing height and body weight were also ascertained at the reference time, and BMI was calculated as the weight (kg) of each participant divided by the square of the height (m^2^) and scaled to reflect a 5 kg/m^2^ increment. Smoking history was defined as never- and ever-smoking.

Participant characteristics by disease status, for all the different exposures are presented in Supplemental Table 2.

### Genotyping, quality assurance/control, and imputation

Details on quality control and genotyping have been previously described [16,29]. Participants were excluded based on genotyping call rates (<97%), heterozygosity, duplicates or next of kin individuals, and discrepancies between self-reported and genotypic sex. We limited analyses to individuals of European ancestry as determined by self-reported race and principal components clustering with 1000 Genomes EUR super-populations [34]. We excluded markers based on missing call rates (>2–5%), departure from Hardy-Weinberg equilibrium (*P* value < 1×10^-4^), and discordant genotype calls within duplicate samples. Genotypes were imputed to the Haplotype Reference Consortium (HRC version r1.1) using the University of Michigan Imputation Server [35,36]. To facilitate data management and analyses, genotypes were converted into a binary format using the BinaryDosage R package (https://cran.r-project.org/web/packages/BinaryDosage). We filtered imputed SNPs based on imputation accuracy of R^2^ > 0.8 and minor allele frequency (MAF) >1%. A total of over 7.2 million SNPs were retained after imputation and quality control. Principal component analysis for population stratification assessment was performed using PLINK1.9 on 30,000 randomly selected imputed SNPs with MAF and R^2^ over 5% and 0.99, respectively [16,29].

### Statistical methods

Directly genotyped SNPs were coded as 0, 1, or 2 copies of the variant allele, whereas for imputed SNPs, the expected number of copies of the variant allele (“dosages”) were used, and log-additive effects were assumed for each SNP [37].

Folate intake variables (μg/d) were coded as sex- and study-specific quartiles prior to modeling. Study-specific associations of folate on CRC outcomes were evaluated using logistic regression models adjusted for age at referent time, sex, and total energy consumption (kcal/d). In sensitivity analyses, models were further adjusted by BMI and smoking status (never/ever), established risk factors for CRC. Estimates were combined using random-effects meta-analysis (Hartung-Knapp) to obtain summary odds ratios (ORs) and 95% confidence intervals (CIs) across studies (when we repeated the analysis using other models [i.e., restricted maximum-likelihood and DerSimonian-Laird], the results were identical) [38]. Heterogeneity was quantified using the inconsistency index (I^2^), and funnel plots were used to identify studies with outlying estimates [39]. Models were fit for all participants and stratified by study design, sex, tumor site (proximal colon, distal colon, and rectum), and regular alcohol use (ever compared with never drinkers) to further investigate any potential effect modification by ethanol. Stratified analyses were also performed by levels of alcohol intake (never drinker, 1–28; ever drinker, >28 g of ethanol daily) and years to CRC diagnosis (<0, 0–1, 1–2, 2–5, >5 years).

Multiplicative statistical interactions for each SNP (G) and folate (E) were investigated using the standard logistic regression analysis (GxE), a 3-degree of freedom (3DF) joint test (which simultaneously tests the marginal associations of G with CRC [D|G], the GxE interaction term, and the G by E correlation [G|E] in the combined case and control set), and a weighted hypothesis 2-step approach [40,41]. In brief, the 2-step approach uses a screening step (step 1) prior to GxE interaction testing (step 2), to decrease multiple testing burden and improve power. In step 1, or screening step, all SNPs are ordered by increasing *P* values based on a combination statistic of D|G and G|E. In step 2, a weighted hypothesis testing approach takes place that orders the SNPs based on lowest *P* value from step 1: SNPs ranked higher in step 1 screening have more lenient alpha thresholds for the interaction test and SNPs ranked lower in step 1 screening have more stringent thresholds such that the overall genome-wide type I error rate for the interaction test is maintained. For primary analyses, a significance threshold of 2×10^-8^ was utilized to account for our use of 3 testing procedures [42,43]. In secondary analyses, statistical interactions were investigated using a 2-degree of freedom (2DF) joint chi-square test (simultaneously testing the D|G association and the GxE interaction term), and a case-only G by E correlation analysis (G|E_case-only_) [44,45]. Interaction loci completely driven by previously known GWAS loci for CRC were filtered out to highlight new loci identified by these methods; these loci (*n* = 203) were analyzed separately, and a Bonferroni correction for multiple comparisons was applied (0.05/203 known loci = 0.00025) [16,20]. Similarly, 3DF and 2DF findings that were only driven by the G|D or G|E association were filtered out. For secondary analyses, an interaction was considered suggestive if it yielded *P* value < 5×10^-8^.

Significant interaction loci were assessed in stratified analyses by study design, sex, tumor site (proximal colon, distal colon, and rectum), and tumor molecular subtypes, including CpG island methylator phenotype (CIMP), microsatellite instability (MSI), and B-Raf proto-oncogene, serine/threonine kinase (*BRAF*) and KRAS proto-oncogene, GTPase (*KRAS*) mutation status. The CIMP, MSI, *BRAF*, and *KRAS* status was determined using specific markers assessed by polymerase chain reaction, sequencing, or immunohistochemistry and was available for a subset of 5342 cases [46]. In the analyses by tumor molecular subtypes, *P* values < 0.05 were considered significant.

The functional role of any statistically significant interaction loci was investigated to establish their potential to regulate gene expression, using functional information from more than 40 genetically diverse human CRC specimens (examining whether the top findings per locus colocalize with transcriptionally active regions) [47]. Potential expression quantitative trait loci (eQTL) associations were explored using the eQTLGen, Genotype-Tissue Expression (GTEx v8), and the University of Barcelona and University of Virginia genotyping and RNA sequencing (BarcUVa-Seq) project dataset, which is comprised of 445 epithelium-enriched healthy colon biopsies. Interaction models using the BarcUVa-Seq data were fit for the gene expression using the standardized gene expression value and with the gene expression broken into groups of 2, 3, or 4 based on the distribution of the expression values [[48], [49], [50]].

All analyses were performed using R (version 4.0.5). The genome-wide interaction scans were performed using the GxEScanR package [51].

### Systematic review of gene-by-folate interactions for CRC

A systematic literature review was performed to summarize commonly studied gene-by-folate interactions. Medline was searched via PubMed (EB), using search terms related to folate, interaction, and CRC to identify primary studies reporting on gene-by-folate interactions up to 11 August, 2022. Systematic reviews and meta-analyses and studies on non-European populations (or of unknown ancestry), nonadvanced adenoma or CRC cases, and gene-gene or folate by other risk factor interaction were excluded.

## Results

### Dietary folate and CRC

**FIGURE 1:**
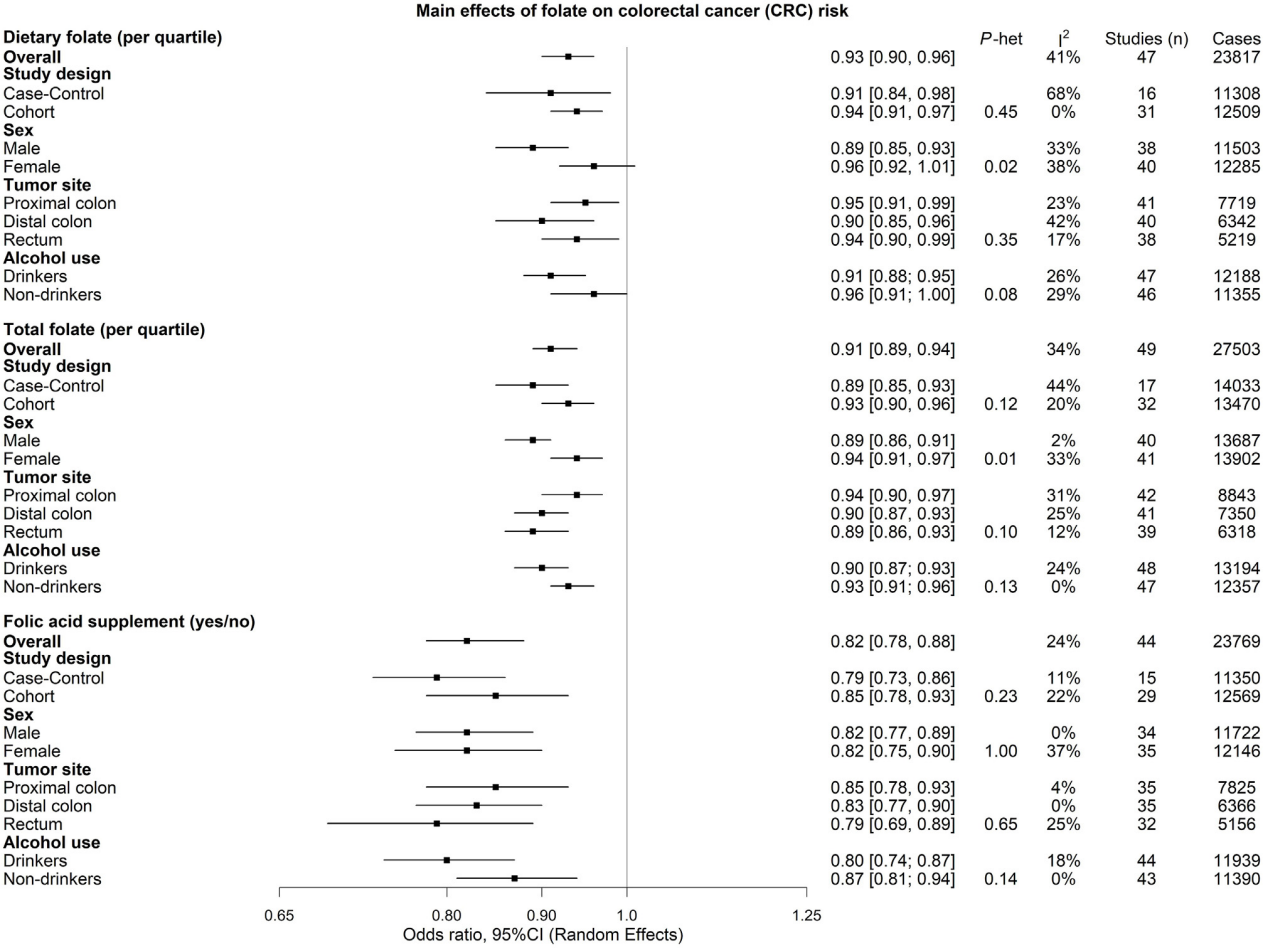
Summary of the random-effects meta-analysis results on the association between folate intake and colorectal cancer risk. CRC, colorectal cancer; I^2^, inconsistency index.

In the pooled analysis an inverse association was found between dietary folate and CRC (OR per quartile higher dietary folate intake: 0.93; 95% CI: 0.90, 0.96; I^2^: 41%). The association was similar by study design, tumor site, and years to CRC diagnosis but was stronger in males than females (*P-*heterogeneity = 0.02) and marginally stronger among ever drinkers compare with never drinkers (*P-*heterogeneity = 0.08) (Figure 1, Supplemental Table 3). Results were similar in sensitivity analyses, further adjusting by BMI and smoking status (Supplemental Figure 2). No significant interaction loci were identified in the primary analyses, namely the standard GxE, the 2-step approach, or the 3DF analysis.

### Total folate and CRC

Total folate was inversely associated with CRC in the pooled analysis (OR per quartile higher total folate intake: 0.91; 95% CI: 0.89, 0.94; I^2^: 34%). The association was similar by study design, anatomical location of the tumor, alcohol use, and years to CRC diagnosis but was stronger in male participants than female (*P*-heterogeneity = 0.01) (Figure 1; Supplemental Table 3). Results were similar in sensitivity analyses, further adjusting by BMI and smoking status (Supplemental Figure 3). Like in the dietary folate analyses, no significant interaction loci were identified in the primary analyses.

### Folic acid supplement use and CRC

For folic acid supplement use, an inverse association with CRC was found in the pooled analysis (OR for users compared with nonusers: 0.82; 95% CI: 0.78, 0.88; I^2^: 24%). Results were similar in the analyses stratified by study design, sex, anatomical location of the tumor, alcohol use, and years to CRC diagnosis (Figure 1, Supplemental Table 3).

**FIGURE 2:**
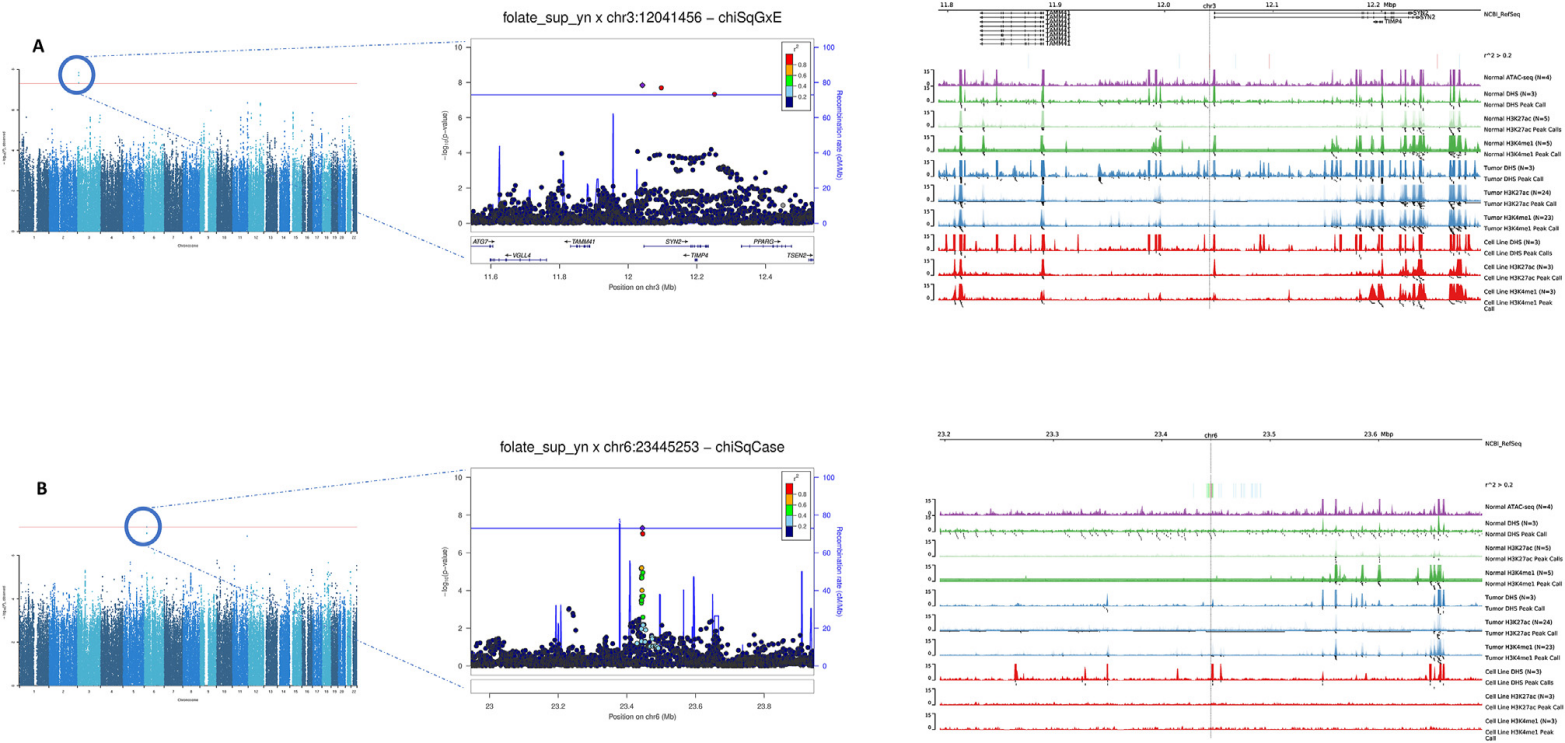
Functional annotation and locus zoom plots of the 2 loci that interacted with folic acid supplement to alter risk of colorectal cancer: (A) variant chr3:12041456 (rs150924902); (B) variant chr6:23445253 (rs1291413). The Manhattan plots (left) provide an overview of the genome-wide interaction scan (GWIS) results, and the locus zoom plots (middle) display regional information centered around the top GWIS findings. The functional annotation plot (right) shows how the top findings per locus colocalize to transcriptionally active regions (peaks), using different markers of chromatin accessibility (ATAC-seq, Assay for Transposase-Accessible Chromatin using sequencing; DHS, DNase I hypersensitive sites; H3K27ac, acetylation of the lysine residue at N-terminal position 27 of the histone H3 protein; H3K4me1, mono-methylation at the 4th lysine residue of the histone H3 protein), and information from genetically diverse human CRC specimens. SYN2, Synapsin II; TAMM41, TAM41 mitochondrial translocator assembly and maintenance homolog; TIMP4, tissue inhibitor of metalloproteinase 4.

An interaction was found in the standard GxE analysis between folic acid supplement and variants in the 3p25.2 locus, near the Synapsin II (*SYN2*)/tissue inhibitor of metalloproteinase 4 (*TIMP4*) region, with rs150924902 (chr3:12041456) being the variant showing the strongest interaction (*P*-interaction = 1.44×10^-8^) (Table 1, Figure 2). In the stratified analyses of folic acid supplement and CRC by genotypes of rs150924902, the association was inverse among those carrying the TT genotype (OR: 0.82; 95% CI: 0.79, 0.86) and positive among those carrying the TA genotype (OR: 1.63; 95% CI: 1.29, 2.05), suggesting a qualitative interaction (Table 2). Too few individuals carried the AA genotype to estimate risk within this group (Table 2). Similar interaction associations were observed in the analyses stratified by study type, sex, and tumor site (Supplemental Table 4). rs150924902 was not associated with colon tissue gene expression in the GTEx v8 data; however, variants in linkage disequilibrium with rs150924902 were eQTLs in other tissues, such as the esophagus muscularis and skeletal muscle (Supplemental Tables 5, 6). There was some evidence for enhanced chromatin accessibility—for variants in the locus of *SYN2* that are correlated with rs150924902—in CRC cell lines and several tissues (Figure 2, Supplemental Table 7). Genes near variant rs150924902 identified via the BarcUVa-Seq dataset were *SYN2*, *TIMP4*, and TAM41 mitochondrial translocator assembly and maintenance homolog (*TAMM41*), of which a significant interaction with folate supplementation was found only for *TIMP4*. The protective effect of supplemental folate intake remained significant for the group with low *TIMP4* expression but was nonsignificant for the group with positive *TIMP4* expression (Supplemental Table 8). In the analyses stratified by molecular subtypes, the interaction was statistically significant only when comparing *BRAF*-mutated cases to controls, but the heterogeneity analysis comparing *BRAF* mutated to nonmutated cases was not statistically significant (Supplemental Table 9).

**TABLE 1:** Summary of the genome-wide interaction loci (*P* < 5×10^-8^) of folic acid supplement intake (yes/no) on colorectal cancer (CRC) risk from the common variant analyses

rsID	Chr	Position	Band	A1	A2	Primary GxE testing		Secondary GxE testing	
						*P*_GxE_	*P*_3DF_	*P*_G|E, case-only_	*P*_2DF_
rs150924902	3	12041456	p25.2	T	A	1.44×10^-8^	1.76×10^-7^	3.36×10^-5^	5.21×10^-8^
rs1291413	6	23445253	p22.3	T	A	7.98×10^-4^	1.80×10^-5^	4.94×10^-8^	3.49×10^-3^

A1, effect allele; A2, reference allele; Band, chromosomal band; Chr, chromosome; *P*_GxE_, *P* value of interaction component for CRC risk (H_0_: β_GxE_=0); *P*_3DF_, *P* value of 3DF test (H_0_: β_GxE_=β_D|G_=δ_G_=0); *P*_G|E, case-only_, *P* value of genetic component for the exposure among cases (H_0_: δ_G_=0); *P*_2DF_, *P* value of 2-degree of freedom (DF) test (H_0_: β_GxE_=β_D|G_=0); Position, base pair position; rsID, single nucleotide polymorphism rs number.

**TABLE 2:** Odds ratios for colorectal cancer stratified by interaction loci genotypes and folic acid supplement use

	chr3:12041456, rs150924902, 3p25.2			chr6:23445253, rs1291413, 6p22.3		
	TT	TA	AA	TT	TA	AA
Folic acid supplement=No	*reference*	0.82 (0.70, 0.95)	0.02 (0, 100)	*reference*	1.02 (0.97, 1.08)	1.08 (1.02, 1.15)
		*P* = 0.010	*P* = 0.35		*P* = 0.38	*P* = 0.015
Folic acid supplement=Yes	0.82 (0.79-0.86)	1.33 (1.11, 1.58)	0.36 (0.04, 3.33)	0.91 (0.85, 0.97)	0.84 (0.79, 0.89)	0.83 (0.77, 0.90)
	*P* = 9.1×10^-21^	*P* = 0.002	*P* = 0.37	*P* = 0.004	*P* = 1.5×10^-8^	*P* = 4.6×10^-6^
**E param by G:**						
Folic acid supplement (yes vs. no)	0.82 (0.79, 0.86)	1.63 (1.29, 2.05)	24.03 (0, 208,977)	0.91 (0.85, 0.97)	0.82 (0.78, 0.87)	0.77 (0.70, 0.83)
	*P* = 9.1×10^-21^	*P* = 4×10^-5^	*P* = 0.49	*P* = 0.004	*P* = 2.1×10^-12^	*P* = 8.7×10^-10^
**G param by E:**						
Folic acid supplement=No	*reference*	0.82 (0.70, 0.95)	0.02 (0, 100.02)	*reference*	1.02 (0.97, 1.08)	1.08 (1.02, 1.15)
		*P* = 0.010	*P* = 1.2×10^-7^		*P* = 0.38	*P* = 0.017
Folic acid supplement=Yes	*reference*	1.61 (1.35, 1.93)	0.44 (0.05, 4.05)	*reference*	0.93 (0.87, 0.99)	0.91 (0.84, 0.99)
		*P* = 0.35	*P* = 0.47		*P* = 0.015	*P* = 0.032
**Counts (Ca/Co):**						
Folic acid supplement=No	14,692/20,921	305/582	0/3	4494/6737	7377/10,583	3126/4186
Folic acid supplement=Yes	8716/13315	288/317	1/3	3064/4405	4343/6743	1597/2486

Estimates represent odds ratios and 95% confidence intervals adjusted for age at baseline, sex, study, genotyping platform, and the first 3 principal components. Number of case/control counts were calculated by imputed genotype probabilities. Ca, cases; Co, controls; E, exposure (folic acid supplement); G, genotype; param, parameterized.

Using the 2-step or the 3DF approach and filtering out the loci that were only driven by the G|D or G|E association, no interaction loci were found. None of the known CRC loci produced significant interactions with folate supplement, or the other folate exposures, after correcting for multiple comparisons (Supplemental Table 10).

In secondary analyses, using the G|E case-only analysis, a statistically significant interaction was found with variants near the 6p22.3 locus, with rs1291413 (chr6:23445253) being the variant that showed the most significant interaction (Table 1, Figure 2). In the analyses of folic acid supplement and CRC stratified by genotypes of rs1291413, there was an inverse association among those carrying the TT genotype (OR: 0.91; 95% CI: 0.85, 0.97), which was stronger among those carrying 1 copy of the A allele (OR: 0.82; 95% CI: 0.78, 0.87), and even stronger among those carrying 2 copies of the A allele (OR: 0.77; 95% CI: 0.70, 0.83) (Table 2). Similar interaction patterns were observed in the analyses stratified by study design, sex, and tumor site (Supplemental Table 4). Variant rs1291413 is located in an intergenic region, and little evidence was found for a regulatory role on gene expression or enhanced chromatin accessibility (Figure 2, Supplemental Tables 5–7). No gene near variant rs1291413 was identified in the BarcUVa-Seq dataset. The association was evident in the analyses comparing nonmutated *BRAF*, nonmutated *KRAS*, individuals with a negative MSI status, and individuals with a positive CIMP status compared with controls, but there was a suggested heterogeneity only by the CIMP status (*P*-interaction = 0.05) (Supplemental Table 9).

### Summary of commonly studied gene-by-folate interactions for CRC

A summary of the previously commonly studied genetic variants for interaction with folate and CRC is presented in Supplemental Table 11. Initial search yielded 479 nonduplicate studies, and after title and abstract screening (EB), 41 were assessed in the full-text screening, of which 13 were included [[23], [24], [25],^27^,^28^,[52], [53], [54], [55], [56], [57], [58], [59]]. The identified studies, which included 196 to 9723 cases, focused on candidate genes in the FOCM pathway, such as methylenetetrahydrofolate reductase (*MTHFR*), 5-methyltetrahydrofolate-homocysteine methyltransferase (*MTR)*, and DNA methyltransferases (DNMTs), or known CRC loci. Most of the studies reported null results or a few weak associations with *P* values for interaction ranging from 0.002 to 0.05.

## Discussion

We investigated the interaction of folate with common variants across the genome, in the largest sample available to date. Novel interactions of folic acid supplement use with common variants near the 3p25.2 locus in relation to CRC risk were found, with functional follow-up analyses providing some evidence of a regulatory role for gene expression. In secondary analysis, a statistically significant interaction was found with variants near the 6p22.3 locus. Furthermore, our pooled analyses, based on the largest sample available to date, including more than 30,000 CRC cases and 40,000 controls, strengthens the overall evidence for an inverse association between folate and CRC risk.

In line with previous studies, the results of our pooled analysis support an inverse association of folate (total, dietary, and supplement) with overall CRC and subtypes (proximal colon, distal colon, and rectal cancer) [8,15]. Furthermore, the estimates were similar for dietary and total folate (including both dietary and supplemental folate), supporting the notion that folate may be effective for CRC prevention even at regular levels of intake that can be achieved through the diet [3]. Mandatory folic acid fortification, like the one implemented in the United States in the mid-1990s, resulted in a substantial increase in bioavailable folate at the population level (2.5-fold increase in the overall US population within a decade) [3,60]. However, in our analysis, study-specific estimates were largely similar across studies from countries with different fortification policies. We found only a slightly larger association for total folate in comparison to dietary folate, which could be attributed to the fact that supplement folate intakes may be more accurately measured [61,62]. Observational studies investigating the association of total folate on CRC have provided estimates comparable to the ones reported in the present study, but randomized controlled trials have reported null results or showed some benefit only among individuals with low folate at baseline [8,10,63]. It must be noted, however, that such trials typically have short follow-up periods (2.3 to 6.7 y), whereas it has been suggested that a longer latency period before CRC diagnosis may exist for the protective effect of folate to manifest [15]. A recently published analysis in the Nurses’ Health Study, with 36 y of follow-up and regular intermediate dietary assessments, provided evidence to support the presence of a latency period of at least 12 y [15]. The researchers found a decrease in CRC risk only 12 to 24 y before diagnosis for total folate (7% to 17% decreased risk per 400 DFE/d, depending on the latency period) and a 9% decrease in CRC risk for synthetic folic acid 16 to 20 y before diagnosis [15].

Two interaction loci with folic acid supplementation were found: rs150924902 (3p25.2) near the *SYN2* region, which emerged in the primary GxE analysis, and rs1291413 (an intergenic variant near the 6p22.3 locus) from a secondary (G|E case-only) analysis. It has been suggested that folate is involved in the synthesis of monoamine neurotransmitters, such as serotonin and dopamine, and experimental studies have shown that folic acid bioavailability might have an impact on their in vivo concentrations [64,65]. Accumulating evidence suggest that deregulation of the serotonergic system might be associated with CRC via its effects on DNA repair and immune response mechanisms [66]. *SYN2* codes for a neuron-specific phosphoprotein that selectively binds to small synaptic vesicles in the presynaptic nerve terminal [67]. Genetic variation in the region of *SYN2* might induce abnormal presynaptic function, thus modulating dopamine and serotonin release [68]. *SYN2* has been previously associated with glioblastoma and prostate cancer, but little evidence is available to date to support a role of *SYN2* in CRC [69,70]. A recent bioinformatics analysis on 3 glioblastoma-related microarray datasets from the Gene Expression Omnibus and The Cancer Genome Atlas identified *SYN2* as a hub gene among the differentially expressed genes, with RNA and protein levels of *SYN2* being downregulated in glioblastoma tissues and high expression of *SYN2* associated with better overall survival among patients with glioblastoma [69]. However, no interaction was found for *SYN2* in relation to folate supplementation using the BarcUVa-Seq data. Interestingly, another cancer-associated gene, *TIMP4*, located within an intron of *SYN2* and transcribed in the opposite direction, was found to interact with folic acid supplement use and alter risk of CRC [71]. Folate availability has been shown to alter plasma homocysteine, and hyperhomocysteinemia might induce an imbalance between the activity of matrix metalloproteinase 9 and TIMP4 [72,73]. TIMP4 is a member of a family of extracellular matrix metalloproteinase inhibitors that has been shown to modulate processes such as cell differentiation, proliferation, and apoptosis [74]. TIMP4 is overexpressed in several cancers, including colorectal, and its expression was found to correlate with longer patient survival (in rectal cancer) [75,76].

Little evidence was found to support a role of locus 6p22.3 in CRC; nevertheless, several novel transcripts are located within 500 kb, with long noncoding RNA ENSG00000289368 (spanning approximately 100 kb on either side of the interaction variant, on the reverse strand) and ENSG00000235743 (approximately 40 kb upwards of the interaction variant) being the closest ones.

To our knowledge, this is the first study to investigate gene-by-folate interactions on a genome-wide scale. Previous studies were small and most included a few hundred cases, and they focused their investigation on a limited number of genetic loci, typically in the FOCM pathway [[22], [23], [24], [25], [26]]. These studies often reported null results or a few nominal associations (i.e., common variants in the DNA methyltransferase 1 [*DNMT1*]*, MTHFR,* 5-methyltetrahydrofolate-homocysteine methyltransferase reductase [*MTRR*]*,* and thymidylate synthetase [*TYMS*] genes), with no adjustment for multiple comparisons [22,24,26]. A potential interaction was reported in a case-control study including 1331 cases and 1501 controls from the PLCO cohort for a variant (rs244072 at locus 20q13.12) in the region of adenosine deaminase (*ADA*) in relation to risk of advanced colorectal adenoma [25]. The association was evident only among individuals in the lowest quartile of dietary folate intake (OR per C allele: 2.33; 95% CI: 1.60, 3.50; *P*: 2.37x10^-5^) and remained statistically significant after a permutation-based adjustment for multiple comparisons [25]. Another study in the WHI Observational Study cohort (including 821 incident CRC case-control matched pairs) that focused on 30 FOCM genes found significant interactions for variants in DNA methyltransferase 3 alpha (*DNMT3A*) (locus 2p23.3) with plasma folate, and *DNMT1* (locus 19p13.2) with red blood cell folate (false discovery rate adjusted q-value was 0.02 for both interaction loci) [23]. Among the most studied loci are C677T (rs1801133) and A1298C (rs1801131), in the region of *MTHFR*, for which an interaction with folate has been suggested [77]. We failed to confirm the above associations, which indicate that they might have been false positive findings attributed to population or exposure-measurement differences, small sample sizes, and inadequate control for false positive findings, all inherent limitations of candidate gene studies. Previous pooled analyses that were based on primary studies participating in the CCFR, CORECT, GECCO consortia, based on a relatively small subset of the participants included in our study (up to 9160 cases), focused the interaction analysis on known risk loci for CRC with dietary folate and only found nominal associations that did not remain after adjustment for multiple comparisons [27,28].

Among the strengths of our study is the large sample size and the comprehensive analyses for overall CRC and subtypes. Furthermore, novel, sophisticated statistical methods were used to investigate interactions with common variants, which limits the multiple-comparison burden. In addition, strict quality-control protocols were applied from data collection to analysis, including harmonization of the folate exposure data, to minimize potential sources of bias. We acknowledge that measurement error in the self-reported exposure assessments may have occurred. Additionally, because most of the primary studies in our analysis did not have follow-up measurements, we used a single measurement of folate intake (and potential confounders) at the reference time for all primary studies; this misses changes that may have occurred over the life course of participants and past exposures (i.e., alcohol intake was based on intake at reference time, capturing most recent intakes; however, in some cases, nondrinkers could be former drinkers). Another limitation is that our analysis is based exclusively on European ancestry populations, which limits the generalizability of the results. No interactions reached statistical significance for dietary and total folate intake, which could be partly attributed to the fact that other exposure variables (such as alcohol) or genetic loci (e.g., folate metabolizing enzymes) may act as additional effect modifiers and to bioavailability differences of the synthetic folic acid compared with natural folate. Folic acid is often found in multivitamins, and we cannot rule out the possibility that the interaction could be due to some other nutrient in multivitamins or a behavior related to multivitamin use. Exploratory analyses to investigate the above hypotheses would be underpowered (substantially limiting the number of cases per stratum) and hence were not included in the present study.

The findings of our study suggest that genetic variation in the region of 3p25.2 might modify the association of folate with CRC risk. Experimental studies and studies incorporating other relevant omics data are warranted to validate this finding.

## Acknowledgments

We are grateful to all the participants who have been part of the project and to the many members of the study teams. For more details, see **Supplementary Acknowledgments &** **Funding**.

### Author contributions

The authors’ responsibilities were as follows—UP, WJG, LH, KKT: designed research; AEK, YL, JM, EB: analyzed the data; EB, KKT wrote the manuscript; AEK, YL, JM, MD, DA, ELB, JWB, SIB, SAB, TDB, HB, AB, ABH, PTC, RCT, GC, TWC, ATC, JCC, DVC, MC, MD, VDO, ND, DAD, JCF, GGG, SBG, MJG, TAH, AH, MH, JRH, ADJ, ESK, TOK, AK, LLM, JPL, LL, BML, BM, SM, VM, NM, PAN, MOS, JO, JRP, NP, BP, AJP, ARP, EAP, JDP, LQ, CQ, GR, ERN, LCS, SLS, AS, MCS, YRS, CMT, DCT, YT, CYU, FvD, BvG, KV, JW, EW, AW, MOW: reviewed and edited the manuscript; KKT, UP, WJG, LH: had primary responsibility for final content; and all authors: read and approved the final manuscript.

### Conflicts of interest

The authors report no conflicts of interest.

### Funding

Konstantinos K Tsilidis was funded by Wereld Kanker Onderzoek Fonds (WKOF) and by World Cancer Research Fund International (WCRF; IIG_FULL_2020_022). Mengmeng Du was funded by P30 CA008748. For more details about the funding of consortia and participating studies, see **Supplementary Acknowledgments &** **Funding**.

### Disclaimer

Where authors are identified as personnel of the International Agency for Research on Cancer/World Health Organization, the authors alone are responsible for the views expressed in this article and do not necessarily represent the decisions, policy, or views of the International Agency for Research on Cancer/World Health Organization.

The authors assume full responsibility for all analyses and interpretation of results. The views expressed here are those of the authors and do not necessarily represent the American Cancer Society or the American Cancer Society – Cancer Action Network.

### Data availability

Data described in the manuscript, code book, and analytic code will be made available upon request pending application and approval to the GECCO consortium.

## Appendix A Supplementary data

Multimedia component1 Multimedia component1

Multimedia component2 Multimedia component2

Multimedia component3 Multimedia component3
