# Supplementary material for: Genome-wide interaction analysis of folate for colorectal cancer risk
Source: Am J Clin Nutr. 2023 Aug 26;118(5):881–91. doi: 10.1016/j.ajcnut.2023.08.010 (PMC10636229; doi:10.1016/j.ajcnut.2023.08.010)
Supplement: Multimedia component1 [file mmc1.pdf]

Genome-wide interaction analysis of folate for colorectal cancer risk

Emmanouil Bouras et al. Online Supplementary Material.

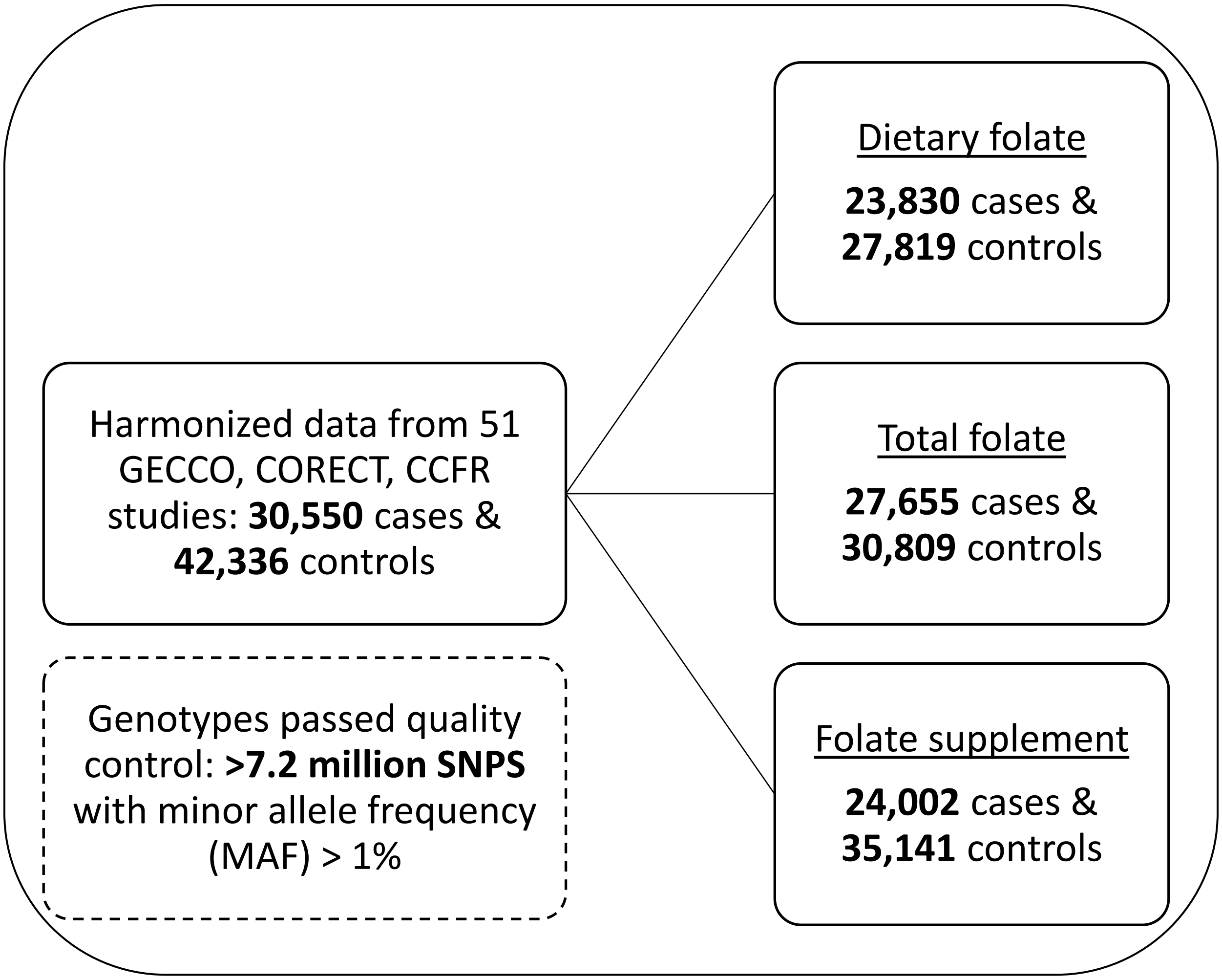

Figure S1. Participant flow chart.

	Source	Control	Case	N	OR	95% CI	p.value
	study_design = Cohort						
	CLUEII	222	206	428	1.03	[0.86; 1.24]	0.73
	CPSII_1	491	487	978	1.04	[0.89; 1.21]	0.63
	CPSII_2	317	308	625	0.98	[0.81; 1.20]	0.87
	EPIC	1986	1716	3702	0.95	[0.89; 1.03]	0.21
	HPFS_1	211	201	412	0.96	[0.80; 1.15]	0.66
	HPFS_2	135	139	274	0.98	[0.78; 1.23]	0.86
	HPFS_3_AD	248	275	523	0.96	[0.81; 1.13]	0.62
	HPFS_4	177	156	333	0.96	[0.78; 1.18]	0.69
	HPFS_5_AD	97	145	242	0.87	[0.68; 1.11]	0.25
	MCCS_1	460	534	994	0.91	[0.79; 1.04]	0.18
	MCCS_2	212	218	430	0.88	[0.72; 1.09]	0.25
	MEC_1	325	306	631	0.92	[0.77; 1.10]	0.35
	MEC_2	89	61	150	0.76	[0.49; 1.17]	0.21
	NHS_1	690	352	1042	0.97	[0.86; 1.10]	0.64
	NHS_2	155	137	292	1.16	[0.92; 1.46]	0.21
	NHS_3_AD	397	487	884	0.97	[0.86; 1.11]	0.67
	NHS_4	263	264	527	0.94	[0.80; 1.11]	0.45
	NHS_5_AD	207	238	445	0.99	[0.84; 1.18]	0.93
	NHSII	15	13	28	1.42	[0.65; 3.12]	0.38
*	PLCO_1_Rematch	292	469	761	0.95	[0.80; 1.14]	0.59
*	PLCO_2	329	417	746	0.82	[0.69; 0.98]	0.03
*	PLCO_3	3049	679	3728	0.90	[0.81; 0.99]	0.04
*	PLCO_4_AD	845	996	1841	0.99	[0.88; 1.11]	0.86
	PPS3	432	67	499	1.22	[0.91; 1.63]	0.18
	PPS4	862	133	995	1.06	[0.85; 1.33]	0.59
	SELECT	266	261	527	0.77	[0.62; 0.95]	0.02
	SMC_COSM	821	503	1324	1.02	[0.89; 1.16]	0.81
*	VITAL	247	231	478	0.94	[0.75; 1.17]	0.56
*	WHI_1_Rematch	507	444	951	0.91	[0.77; 1.07]	0.26
*	WHI_2	982	957	1939	0.89	[0.79; 1.00]	0.04
*	WHI_3	556	544	1100	0.90	[0.77; 1.06]	0.22
	Total (fixed effect)				0.95	[0.92; 0.98]	
	Total (random effects)				0.95	[0.92; 0.98]	
	Heterogeneity: χ^2_{30} = 23.97 (P = .77), I^2 = 0%						
	study_design = Case-Control						
	CCFR_1	473	360	833	1.07	[0.92; 1.25]	0.36
	CCFR_3	579	751	1330	0.95	[0.85; 1.08]	0.45
	CCFR_4	205	138	343	0.93	[0.74; 1.17]	0.53
	Colo23	107	75	182	0.74	[0.51; 1.07]	0.11
	CRCGEN	937	772	1709	0.87	[0.79; 0.95]	0.00
	DALS_1	701	696	1397	0.82	[0.73; 0.92]	0.00
	DALS_2	459	396	855	0.93	[0.79; 1.09]	0.35
	HawaiiCCS_AD	528	80	608	0.85	[0.64; 1.13]	0.26
	Kentucky	891	769	1660	0.89	[0.78; 1.01]	0.07
	LCCS	661	1150	1811	0.93	[0.84; 1.03]	0.16
	MECC_1	419	404	823	0.90	[0.77; 1.05]	0.19
	MECC_2	885	978	1863	1.03	[0.93; 1.14]	0.57
	MECC_3	2554	3221	5775	0.93	[0.87; 0.99]	0.02
	NCCCSI	460	237	697	0.72	[0.61; 0.86]	0.00
	NCCCSII	650	555	1205	0.82	[0.71; 0.95]	0.01
	NFCCR_2	448	179	627	1.31	[1.09; 1.58]	0.00
	Total (fixed effect)				0.92	[0.89; 0.95]	
	Total (random effects)				0.92	[0.85; 0.98]	
	Heterogeneity: χ^2_{15} = 39.74 (P < .001), I^2 = 62%						
	Total (fixed effect)				0.94	[0.92; 0.96]	
	Total (random effects)				0.94	[0.91; 0.97]	
	Heterogeneity: χ^2_{46} = 65.82 (P = .03), I^2 = 30%						
	Test for subgroup differences (fixed effect): χ^2_1 = 2.11 (P = .15)						
	Test for subgroup differences (random effects): χ^2_1 = 0.99 (P = .32)						

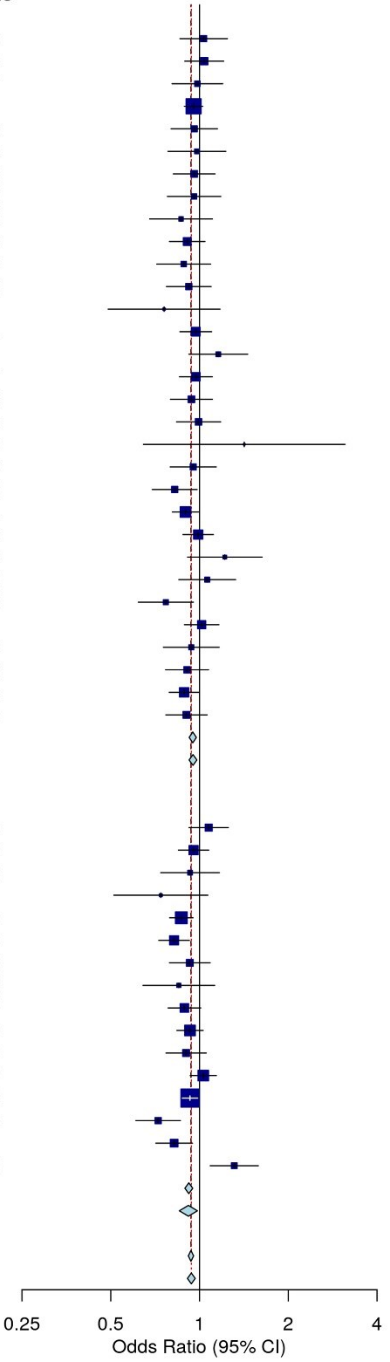

Figure S2. Meta-analysis of dietary folate on risk of colorectal cancer (CRC), stratified by study design. Per study models adjusted for age at referent time, sex, total energy consumption (kcal/day), body mass index (BMI), and smoking status (never/ever). In studies marked with an asterisk (*****) [namely, Prostate, Lung, Colorectal, & Ovarian Cancer Screening Trial (PLCO), VITamin And Lifestyle Study (VITAL), and Women's Health Initiative (WHI)] times of enrollment overlapped or followed the period of folic acid fortification (1996-1998), which was accounted for when calculating dietary folate intake.

	Source	Control	Case	N	OR	95% CI	p.value
	**study_design = Cohort**						
	CLUEII	252	265	517	1.02	[0.87; 1.20]	0.78
	CPSII_1	500	497	997	0.89	[0.79; 1.00]	0.05
	CPSII_2	323	309	632	0.97	[0.84; 1.12]	0.71
	EPIC	1986	1721	3707	0.95	[0.89; 1.02]	0.19
	HPFS_1	220	218	438	0.75	[0.62; 0.90]	0.00
	HPFS_2	153	162	315	0.99	[0.80; 1.21]	0.90
	HPFS_3_AD	273	291	564	0.96	[0.82; 1.11]	0.56
	HPFS_4	186	169	355	0.91	[0.75; 1.10]	0.32
	HPFS_5_AD	101	151	252	0.92	[0.73; 1.17]	0.51
	MCCS_1	461	534	995	0.87	[0.75; 1.02]	0.08
	MCCS_2	212	218	430	0.80	[0.64; 1.01]	0.07
	MEC_1	337	320	657	0.96	[0.83; 1.11]	0.60
	MEC_2	89	64	153	0.83	[0.59; 1.18]	0.30
	NHS_1	740	379	1119	0.94	[0.83; 1.05]	0.27
	NHS_2	176	155	331	0.96	[0.78; 1.18]	0.70
	NHS_3_AD	409	501	910	0.98	[0.87; 1.11]	0.79
	NHS_4	292	295	587	0.92	[0.79; 1.07]	0.28
	NHS_5_AD	217	247	464	0.95	[0.79; 1.13]	0.54
	NHSII	99	105	204	1.28	[1.00; 1.65]	0.05
*	PLCO_1_Rematch	319	526	845	0.96	[0.85; 1.09]	0.56
*	PLCO_2	361	456	817	0.89	[0.79; 1.00]	0.05
*	PLCO_3	3357	736	4093	0.93	[0.87; 1.00]	0.04
*	PLCO_4_AD	923	1090	2013	0.95	[0.87; 1.02]	0.17
	PPS3	450	68	518	0.99	[0.78; 1.25]	0.91
	PPS4	910	143	1053	1.07	[0.87; 1.33]	0.50
	SELECT	266	261	527	0.79	[0.66; 0.94]	0.01
	SMC_COSM	821	503	1324	1.02	[0.89; 1.16]	0.82
*	VITAL	267	255	522	0.91	[0.78; 1.07]	0.28
*	WHI_1_Rematch	507	444	951	1.04	[0.92; 1.19]	0.51
*	WHI_2	985	961	1946	0.91	[0.83; 0.99]	0.03
*	WHI_3	556	545	1101	0.90	[0.79; 1.01]	0.08
	Total (fixed effect)				0.94	[0.92; 0.96]	
	Total (random effects)				0.94	[0.91; 0.97]	
	Heterogeneity: χ^2_{30} = 30.6 (P = .44), I^2 = 2%						
	**study_design = Case-Control**						
	CCFR_1	901	944	1845	0.94	[0.86; 1.03]	0.20
	CCFR_3	608	845	1453	0.94	[0.85; 1.05]	0.26
	CCFR_4	588	1048	1636	0.85	[0.77; 0.94]	0.00
	Colo23	107	75	182	0.81	[0.60; 1.10]	0.18
	CRCGEN	972	839	1811	0.90	[0.83; 0.99]	0.03
	DALS_1	701	696	1397	0.93	[0.84; 1.03]	0.19
	DALS_2	459	396	855	0.90	[0.79; 1.03]	0.14
	HawaiiCCS_AD	530	80	610	0.69	[0.55; 0.86]	0.00
	Kentucky	899	778	1677	0.89	[0.81; 0.97]	0.01
	LCCS	669	1361	2030	0.99	[0.90; 1.10]	0.89
	MECC_1	465	432	897	0.92	[0.81; 1.05]	0.20
	MECC_2	982	1020	2002	0.93	[0.85; 1.01]	0.09
	MECC_3	2641	3306	5947	0.93	[0.88; 0.98]	0.01
	NCCCSI	461	241	702	0.77	[0.66; 0.90]	0.00
	NCCCSII	650	558	1208	0.86	[0.77; 0.95]	0.01
	NFCCR_2	448	179	627	1.03	[0.86; 1.23]	0.75
	Total (fixed effect)				0.91	[0.89; 0.93]	
	Total (random effects)				0.90	[0.86; 0.94]	
	Heterogeneity: χ^2_{15} = 21.34 (P = .13), I^2 = 30%						
	Total (fixed effect)				0.92	[0.91; 0.94]	
	Total (random effects)				0.92	[0.90; 0.95]	
	Heterogeneity: χ^2_{46} = 54.59 (P = .18), I^2 = 16%						
	Test for subgroup differences (fixed effect): χ^2_1 = 2.65 (P = .10)						
	Test for subgroup differences (random effects): χ^2_1 = 2.38 (P = .12)						

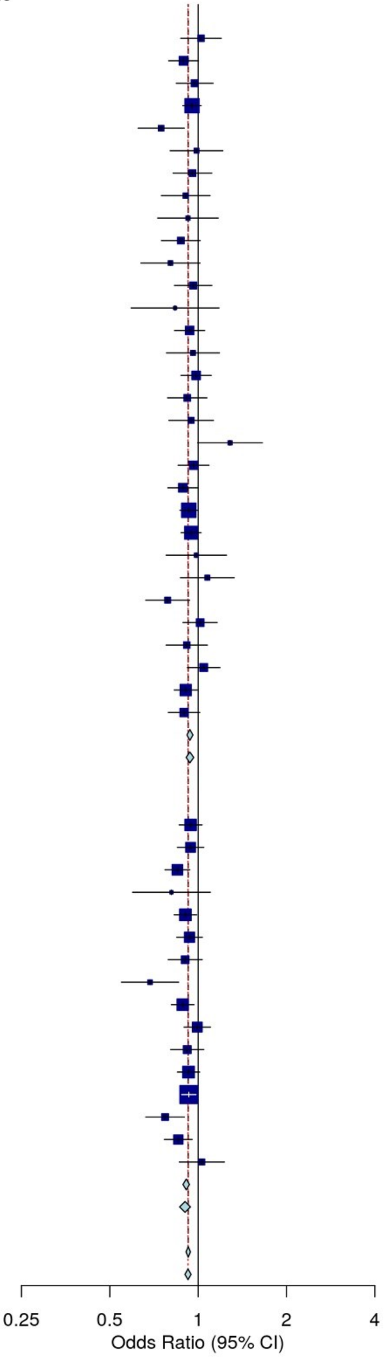

Figure S3. Meta-analysis of total folate on risk of colorectal cancer (CRC), stratified by folic acid fortification status. Per study models adjusted for age at referent time, sex, total energy consumption (kcal/day), body mass index (BMI), and smoking status (never/ever). In studies marked with an asterisk (*) [namely, Prostate, Lung, Colorectal, & Ovarian Cancer Screening Trial (PLCO), VITamin And Lifestyle Study (VITAL), and Women’s Health Initiative (WHI)] times of enrollment overlapped or followed the period of folic acid fortification (1996-1998), which was accounted for when calculating dietary folate intake.
